# Trivalent Dopant Size Influences Electrostrictive Strain in Ceria Solid Solutions

**DOI:** 10.1021/acsami.0c20810

**Published:** 2021-04-22

**Authors:** Maxim Varenik, Juan Claudio Nino, Ellen Wachtel, Sangtae Kim, Sidney R. Cohen, Igor Lubomirsky

**Affiliations:** † Department of Molecular Chemistry and Materials Science, Weizmann Institute of Science, Rehovot 7610001, Israel; ‡ Department of Materials Science and Engineering, University of Florida, Gainesville, Florida 32611, United States; § Department of Materials Science and Engineering, University of California, Davis, Davis, California 95616, United States; ∥ Dept. Chemical Research Support, Weizmann Institute of Science, Rehovot 7610001, Israel

**Keywords:** electrostriction, nanoindentation, anelasticity, doped ceria, Young’s modulus

## Abstract

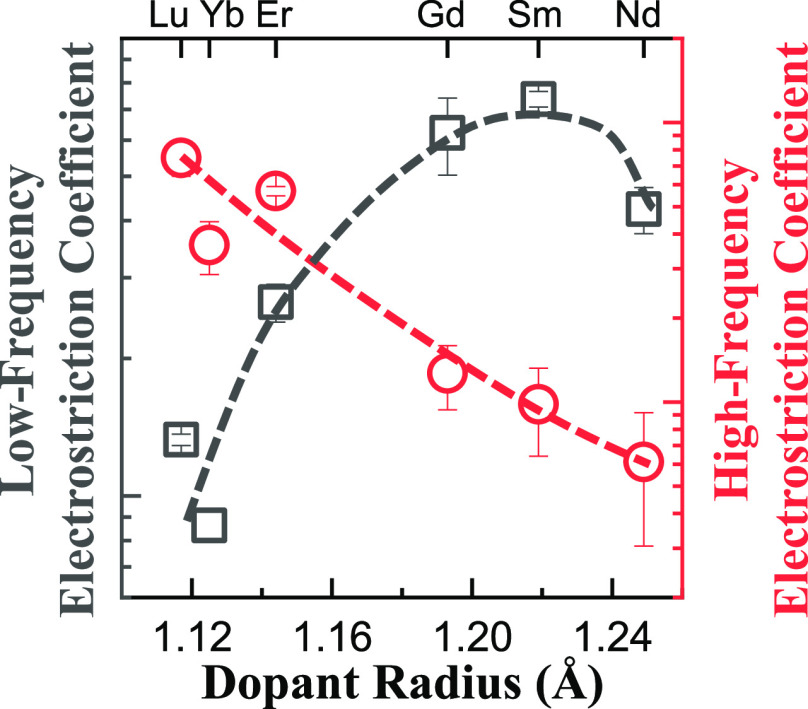

The technologically important frequency range for the application of electrostrictors and piezoelectrics is tens of Hz to tens of kHz. Sm^3+^- and Gd^3+^-doped ceria ceramics, excellent intermediate-temperature ion conductors, have been shown to exhibit very large electrostriction below 1 Hz. Why this is so is still not understood. While optimal design of ceria-based devices requires an in-depth understanding of their mechanical and electromechanical properties, systematic investigation of the influence of dopant size on frequency response is lacking. In this report, the mechanical and electromechanical properties of dense ceria ceramics doped with trivalent lanthanides (RE_0.1_Ce_0.9_O_1.95_, RE = Lu, Yb, Er, Gd, Sm, and Nd) were investigated. Young’s, shear, and bulk moduli were obtained from ultrasound pulse echo measurements. Nanoindentation measurements revealed room-temperature creep in all samples as well as the dependence of Young’s modulus on the unloading rate. Both are evidence for viscoelastic behavior, in this case anelasticity. For all samples, within the frequency range *f* = 0.15–150 Hz and electric field *E* ≤ 0.7 MV/m, the longitudinal electrostriction strain coefficient (|*M*_33_|) was 10^2^ to 10^4^-fold larger than expected for classical (Newnham) electrostrictors. However, electrostrictive strain in Er-, Gd-, Sm-, and Nd-doped ceramics exhibited marked frequency relaxation, with the Debye-type characteristic relaxation time τ ≤ 1 s, while for the smallest dopants—Lu and Yb—little change in electrostrictive strain was detected over the complete frequency range studied. We find that only the small, less-studied dopants continue to produce useable electrostrictive strain at the higher frequencies. We suggest that this striking difference in frequency response may be explained by postulating that introduction of a dopant induces two types of polarizable elastic dipoles and that the dopant size determines which of the two will be dominant.

## 1 Introduction

Undoped and aliovalent cation-doped ceria has a wide range of applications as intermediate-temperature (IT) oxygen ion conductors^1,2^ and in the field of catalysis.^3^ Such broad technological interest has motivated detailed experimental and theoretical studies of ceria-based materials, many of which have been comprehensively reviewed in refs (4) and (5). Superior performance of ceria-based materials stems, at least in part, from charge-compensating oxygen vacancies induced by aliovalent doping.^2,6^ X-ray absorption spectroscopy and micro-Raman spectroscopy measurements (described in the above-cited reviews and in articles referenced therein) provide details for the central role that the lattice defect chemistry and structure may play in determining the complex electrostrictive behavior of doped and/or reduced ceria.

For Gd-doped ceria, a number of anomalies in mechanical and electromechanical properties have been reported. These anomalies include anelasticity (time-dependent elastic moduli in an equilibrium solid),^7−9^ dependence of Poisson’s ratio on the strain magnitude,^10,11^ spontaneous volume expansion over time,^12,13^ hysteresis of the lattice parameter during thermal cycling,^12,14^ and an unusually large electrostrictive response.^9,15,16^ Two types of measurements reveal particularly striking anomalies: nanoindentation (NI) and electromechanical strain. Room-temperature NI measurements of Gd- and Sm-doped ceria ceramics revealed the presence of primary creep,^8,17−19^ as well as dependence of calculated Young’s modulus on the unloading rate. It is important to note that for a material like ceria, in which dislocation movement at room temperature is not likely, primary creep deformation is a clear sign of viscoelastic (of which anelasticity is one example) behavior. The electrostriction strain coefficient, measured in both thin films and ceramics at room temperature, is at least 2 orders of magnitude higher^9,15,18−27^ than expected from the empirical scaling law proposed by Newnham and co-workers.^28,29^

Detailed studies of the electrostriction effect in Gd- and Sm-doped ceria revealed two important features.^9,20^ First, for dopant concentrations ≤10–15 mol %, the longitudinal electrostriction strain coefficient undergoes ∼10^2^-fold reduction in magnitude as a function of frequency, that is, ≈10^–16^ m^2^/V^2^ for *f* < 1 Hz to ≈10^–18^ m^2^/V^2^ for *f* > 100 Hz. Both values are much larger than predicted from the scaling law obeyed by diverse, classical (Newnham^28,30^) electrostrictors (≈10^–20^ m^2^/V^2^). Second, increasing the dopant concentration results in a marked decrease in the quasistatic electrostriction strain coefficient, while the high-frequency and low-frequency electrostriction strain coefficients are both ≈10^–18^ m^2^/V^2^.^20,23,24^ The microscopic features responsible for such strikingly large electrostrictive strain and room-temperature anelasticity^7^ are still a subject of considerable debate. Recent theoretical studies,^31−34^ high-energy resolution X-ray absorption spectroscopy,^35^ and reverse Monte Carlo modeling constrained by extended X-ray absorption fine structure spectroscopy (EXAFS) and X-ray diffraction (XRD) data,^36^ with supercell (5 × 5 × 5 fluorite unit cells), have provided additional key important information and insights. EXAFS probes cation-specific, short-range—that is, NN and NNN—distances, while powder XRD is insensitive to non-correlated local lattice distortions. These simulations revealed that at room temperature, the equilibrium distributions of cation–oxygen and cation–cation distances are bimodal and strongly influenced by the crystal radius of the dopant cations and oxygen vacancies positioned at random in the fluorite lattice. The dopants studied were Sm [III, *r*_crystal_ = 1.22 Å for coordination number (CN) = 8], which is larger than the host cation, Ce(IV) (*r*_crystal_ = 1.11 Å for CN = 8), and Y (III, *r*_crystal_ = 1.16 Å for CN = 8). It is of course well-documented that with increasing trivalent dopant concentration, the lattice average CN for the cation decreases from 8. However, for the largest dopant concentration considered in ref (33), 20 mol %, the average value of CN = 7.6. Values of the crystal radius (uniformly ∼0.14 Å larger than the more familiar ionic radius) were taken from ref 37. The doping level was limited to 20 mol % to avoid the double fluorite phase. Such point defect-derived local distortion is viewed as giving rise to the formation of elastic dipoles (see Supporting Information, Section S2) with a broad distribution of dipole strengths and relaxation times.^8,12−14,17,26^ On the basis of density functional theory (DFT) modeling of reduced ceria,^31^ Qi and co-workers have suggested that charge disproportionation in the vicinity of oxygen vacancies induces strongly anisotropic local strain, forming a polarizable elastic dipole which contributes to anelastic behavior. The architecture of the putative polarizable elastic dipoles remains incompletely characterized, as does the frequency dependence of the electrostrictive strain coefficient.

Local lattice distortion in aliovalent-doped ceria appears to correlate with the crystal radius of the dopant.^36^ Therefore, we expect that measuring electrostrictive strain as a function of dopant size should provide information on the nature of the elastic dipole’s dominating response at high and low electric field frequencies. In the current work, investigation of mechanical moduli, electrostrictive strain, and anelastic response under NI was extended to Lu-, Yb-, Er-, and Nd-doped ceria ceramics. Dopant concentration was fixed at 10 mol %, and to minimize the influence of the preparation procedures on the sample properties, all ceramics used in this study (RE_0.1_Ce_0.9_O_1.95_, RE = Lu, Yb, Er, Gd, Sm, and Nd) were prepared with the same protocol. The choice of the 10 mol % dopant was determined by the need to remain well within the fluorite phase^38−40^ while generating a sufficient number of point defects, including 2.5% charge-compensating oxygen vacancies.

## 2 Experimental Section

### 2.1 Sample Preparation

Solid solutions RE_0.1_Ce_0.9_O_1.95_, with RE = Lu, Yb, Er, Gd, Sm, or Nd, were synthesized via conventional solid-state reactions and procedures as previously described.^41^ Briefly, RE_2_O_3_ and CeO_2_ powders (both 99.99% purity) were ball-milled, dried, and calcined at 1450 °C for 10 h. Up to 2 wt % binder (PVA dissolved in deionized water) was added to the powders, and cylindrical pellets were formed in metal dies by uniaxial pressing. It is important to note that the presence of PVA does not modify mechanical properties, particularly since the green ceramics undergo sintering.^8^ The pellets were then subjected to an isostatic pressure at 250 MPa for 3 min, followed by sintering at 1600 °C for 10 h. Sintered pellet dimensions were 6–8 mm diameter and ∼1 mm thickness. The porosity of all sintered pellets was deduced from the mass density measured by the conventional Archimedes technique. Pellets were polished and the top and bottom faces were made parallel with silicon carbide polishing papers (up to 1600 mesh). The silicon carbide residue was removed by 30 min of washing with 100% ethanol in an ultrasonic bath. Prior to electromechanical measurements, all samples were heated at 500 °C for 5 h in a pure oxygen atmosphere to compensate for possible oxygen loss during sintering.

### 2.2 X-ray Diffraction

XRD was used to determine the phase of the ceria solid solution ceramics. A theta–theta diffractometer was employed—Ultima III (Rigaku, Japan). The operating mode was Bragg–Brentano with variable beam divergence, 2θ angle range 20–120°. Jade_Pro (MDI, CA) software provided data analysis.

### 2.3 Ultrasound Time-of-Flight (Ultrasound Pulse Echo) Measurements

Shear (transverse, VS) and longitudinal (VL) sound velocities were determined with an accuracy better than 0.25% (pellet height measured with the uncertainty ≤ 0.15%) with ultrasound time-of-flight (USTOF) instrumentation and protocol as described in ref (42) and in previous reports.^8,43−45^ USTOF was measured using transducers coupled directly to the pellets with high-viscosity commercial honey without external force. Correction for porosity < 4 vol % was performed as described previously^8,30^ (see Supporting Information, Section S1). Typical pulse echo decay profiles are provided in Figure S1, and sound velocities, uncorrected for porosity, are reported in Figure S2.

### 2.4 NI Measurements

NI measurements were carried out under ambient conditions using a KLA-Tencor-XP instrument with a Berkovich indenter. Measurements were performed at ≥10 locations on each pellet at depths between 700 and 1100 nm to reduce the effect of heterogeneity while keeping the area sampled by the indentation small. Measurements were performed using a protocol described previously^15,17,30^ with a trapezoidal load–hold–unload cycle. The “fast” measurement consisted of loading at a rate of 15 mN/s to a maximum load of 135 mN, a load-hold time of 8 s at the maximum load, and load-removal at 5 mN/s. We have previously observed that for Sm-doped ceria ceramics, more rapid load removal produced a negative slope upon indenter retraction, thereby rendering the results unsuitable for modulus calculation using the Oliver–Pharr protocol.^8^ The “slow” measurement consisted of loading at a rate of 0.15 mN/s to a maximum load of 135 mN, a load-hold time of 30 s at the maximum load, and an unloading rate of 0.15 mN/s. Young’s modulus (*E*) was determined from the initial slope of the unloading phase of the cycle using Oliver–Pharr analysis for elastic solids.^46,47^ Loading and unloading curves selected for calculations were smooth, without “pop-ins” or instabilities. Thermal drift rates measured in separate experiments at 90% unloading were less than 0.1 nm/s and did not significantly influence the results. Typical indenter displacement curves are provided in Figure S3.

### 2.5 Electrostrictive Strain

Longitudinal (i.e., parallel to the applied electric field) electrostrictive strain, *u*_33_, was measured with instrumentation described previously.^9,20,30^ Briefly, the ceramic pellet was inserted between two stainless-steel electrodes, the top electrode being spring-loaded. Voltage was applied using a Keithley 3390 waveform generator and a Trek 610E amplifier. A pushrod was used to transfer displacement from the electrodes to a proximity sensor (capacitance, CPL190 Lion); the signal from the proximity sensor was read with a lock-in amplifier (DSP 7265). Longitudinal electrostrictive strain is calculated as the ratio between the displacement and the original thickness of the ceramic pellets as measured with a Mitutoyo (193–111, ±2 μm) screw gauge. Measurements were performed under ambient conditions (24 ± 2 °C, relative humidity 20–55%). Commercial samples of Pb(Mg_1/3_Nb_2/3_)O_3_−PbTiO_3_ (PMN-PT)with silver metal contacts (TRS Technologies) and a 100-cut quartz single crystal without additional sputtered metal contacts were used for calibration of the measurement setup. Values matching the literature data were obtained: *M*_33_ = (3.5 ± 0.5)·10^–16^ m^2^/V^2^ for PMN-PT and *d*_33_ = 2.3 ± 0.2 pm/V for the quartz crystal within the frequency range 150 mHz to 1 kHz.

## 3 Results and Discussion

### 3.1 Structure

**Figure 1:**
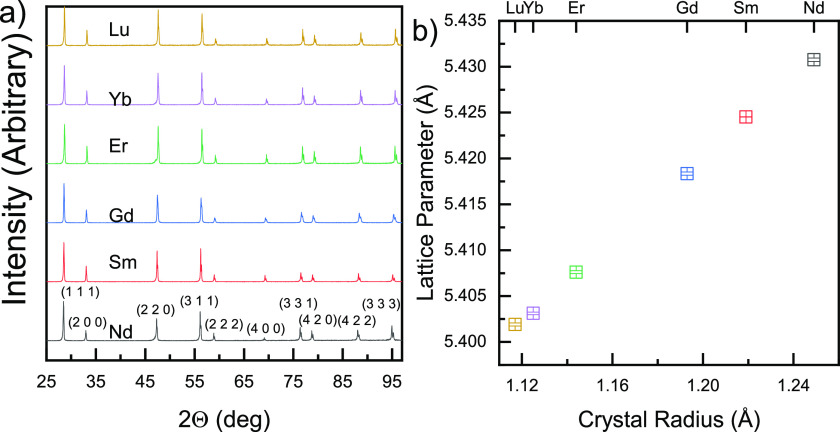
(a) XRD patterns (with variable beam divergence as a function of the scattering angle) measured at room temperature on the ceramic surface. Correcting the XRD peak intensities for fixed beam divergence with Jade_PRO software and comparing them to standard powder patterns in the Inorganic Crystal Structure Database confirm that the ceramics are single-phase and not textured. This comparison cannot be made with variable beam divergence, which is our usual operating protocol, as is noted in the Experimental Section. (b) Lattice constants for RE_0.1_Ce_0.9_O_1.95_ (RE = Lu, Yb, Er, Gd, Sm, and Nd) were calculated by linear regression after indexing 10 diffraction peaks according to *Fm*3̅*m* symmetry. The lattice constant of un-doped fully oxidized ceria under ambient conditions is 5.411 ± 0.001 Å. Crystal radii are due to Shannon^37^ for valence III and CN 8.

**Figure 2:**
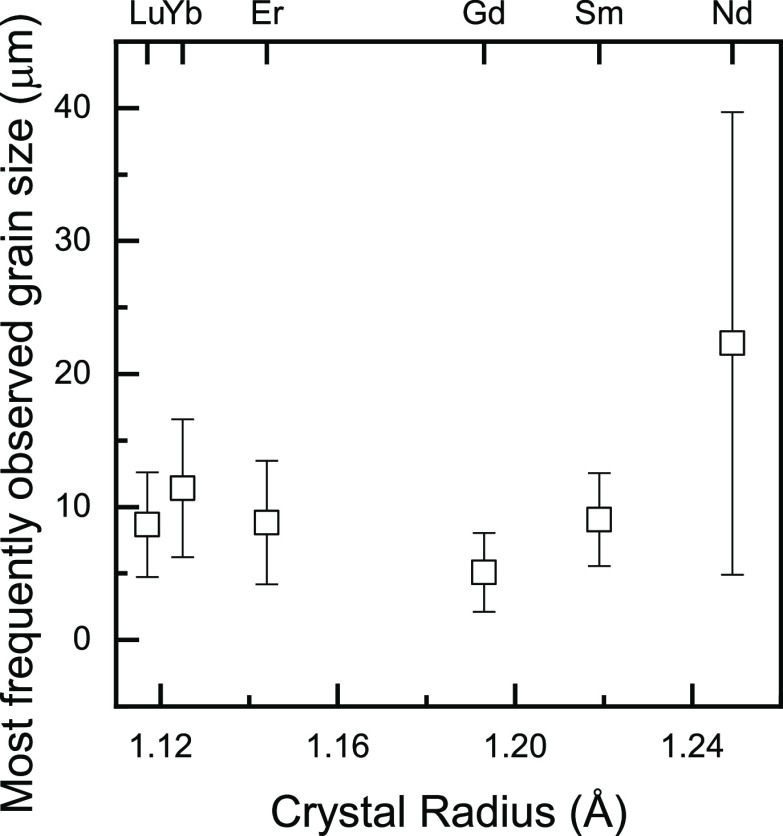
Most frequently observed grain size in SEM images according to the lineal intercept method^48^ for a log–normal distribution as a function of the crystal radius of the trivalent dopant for CN 8. Nd doping produces the broadest size distribution (standard deviation, error bars).

XRD patterns of the ceria solid solution ceramic pellets, doped with 10 mol % trivalent lanthanides (Lu, Yb, Er, Gd, Sm, or Nd), can be indexed as an untextured cubic fluorite polycrystal (*Fm*3̅*m*, Figure 1a). The lattice parameter increases linearly with increasing crystal radius of the dopant^37^ (Figure 1b). SEM (Zeiss Sigma 500) images of pellet circumferential surfaces revealed that the grain sizes, as estimated by the lineal intercept method,^48^ follow a log–normal distribution; the median observed sizes on the SEM images are Lu-6 μm; Yb-7 μm; Er-6 μm; Gd-3 μm; Sm-6 μm; and Nd-14 μm (Figure 2). Sm-doped ceria displays the narrowest size distribution and Nd the broadest. The corrected median grain size is obtained from these values following multiplication by Mendelson’s factor (×1.56),^48^ which accounts for the fact that not all grain diameters are fully measurable at the pellet surface. Although in the case of perovskite piezo-ceramics, grain size is thought to play a role in modulating the electromechanical strain response,^49^ for aliovalent-doped ceria, no obvious influence of grain size or size distribution (Figure 2) has been observed. Published electrostriction coefficients of thin films and bulk samples are very similar, although grain sizes may differ by up to 3 orders of magnitude.^8,16,18,19,21−25^

### 3.2 Measurement of Elastic Moduli

**Figure 3:**
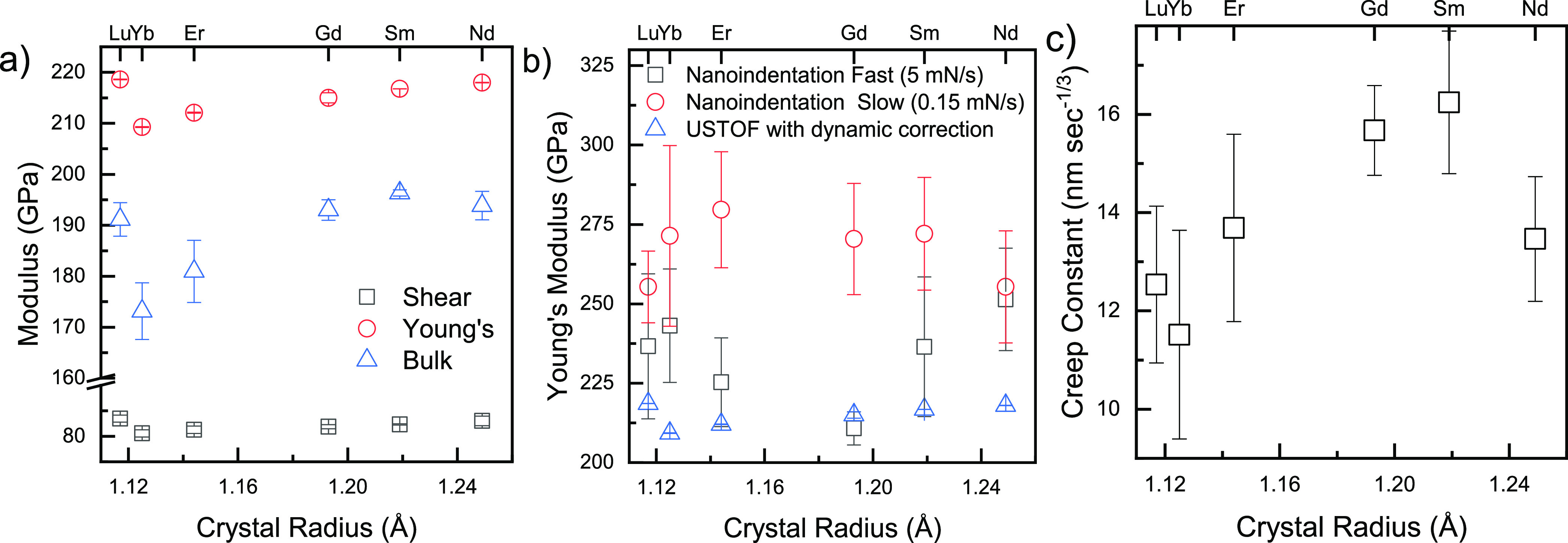
(a) Young’s, shear, and bulk moduli, calculated from ultrasound pulse echo measurement of sound velocity and corrected for porosity, as a function of the lanthanide dopant crystal radius. Values of the crystal radius assume CN = 8.^37^ (b) Young’s modulus calculated using the Oliver and Pharr NI protocol^8^ for slow (0.15 mN/s) and fast (5 mN/s) unloading rates. Values obtained by sound velocity measurements are included for comparison. (c) Primary creep constant calculated from NI measurements, fast loading rate 150 mN/s, 8 s maximum load (135 mN) hold. Error bars are standard deviation from the mean of ≥10 room-temperature measurements at different locations on the surface of the same pellet.

USTOF measurements displayed more than 12 reflections on echo/time plots (Supporting Information, Figure S1), testifying to the absence of fissures and microcracks in the ceramic pellets. Following sintering, the measured mass density of all pellets was >95% of the theoretical (XRD) value. This is sufficient to correct the elastic moduli deduced from the USTOF^8,42^ for residual porosity using the dynamic correction equations^50,51^ (see Supporting Information, Section S1). Following correction for porosity, the elastic moduli show little statistically significant difference among the dopants (Figure 3a), with, for example, a Young’s modulus of ∼219 GPa for the smallest (Lu) and the largest (Nd) dopant. However, the values for all moduli are lower than those measured previously for undoped ceria. This observation is attributed to the weakening of interatomic bonds due to the introduction of charge-compensating oxygen vacancies.^8,43^

The values of Young’s modulus calculated from NI measurements using the Oliver–Pharr protocol with a slow unloading rate (0.15 mN/s) were consistently larger than those obtained from USTOF measurements (Figure 3a), and the uncertainty was much larger. Time-dependent, yet linear, response to externally applied mechanical stress is an indication of viscoelasticity; in the special case of an equilibrium solid, the response is anelastic, that is, completely reversible, given sufficient time. Although anelastic materials may, in principle, display time-dependent moduli under hydrostatic stress, that is not true for aliovalent-doped ceria, where point defects produce low symmetry lattice distortions. If isotropic stress is applied, there will be no energetic advantage for reconfiguration of the defect; however, if the stress is anisotropic, then reorientation of the defect, considered as an elastic dipole, will occur in order to minimize its interaction energy. This reconfiguration of the defect will produce a time-dependent anelastic response (see Section S3 in the Supporting Information). Additional confirmation for the presence of anelasticity comes from the fact that at room temperature, all samples exhibit creep, that is, displacement under a constant load (Figure 3c). As observed in the case of Gd-doped ceria^17−19^ and (Y,Nb)-stabilized Bi_2_O_3_,^30^ the primary creep follows the time–displacement dependence^52^
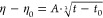
1where η_0_ is the displacement at the beginning of the load-hold stage (*t*_0_) and *A* is the creep constant.^53^ The statistical uncertainty of the NI-derived values of the Young’s modulus is too large to enable extraction of any unambiguous dependence on the dopant size. However, one-way analysis of variance (ANOVA) statistical analysis does allow us to conclude that the primary creep constants of Gd- and Sm-doped ceria ceramics (Figure 3c) are equivalent at the 0.05 confidence level and both are larger than the creep constants of the other four dopants, tested pairwise.

### 3.3 Electrostrictive Strain

**Figure 4:**
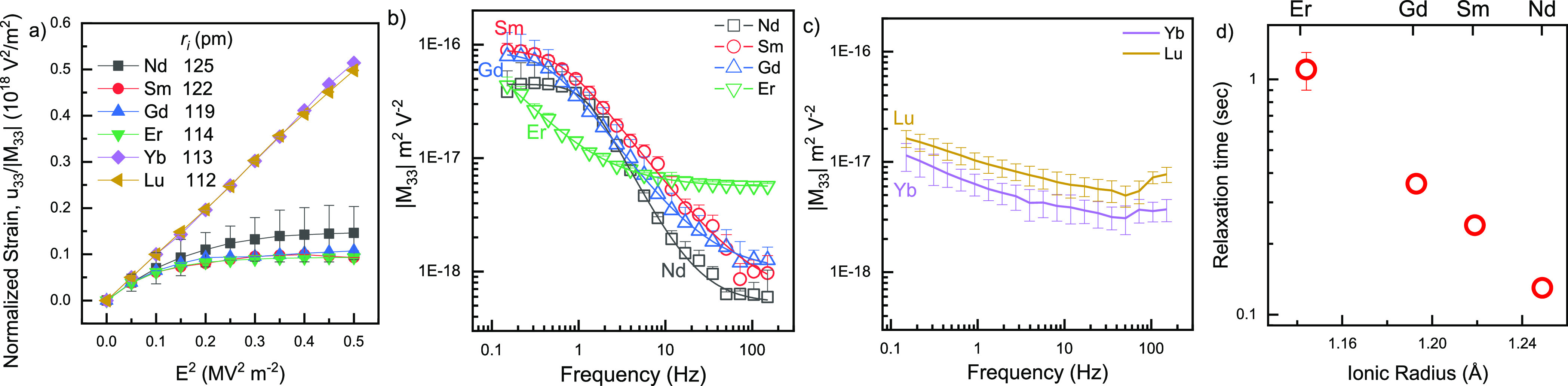
(a) Normalized longitudinal strain as a function of the square of the electric field. The strain was normalized to the electrostriction coefficient, |*M*_33_|, at *f* = 0.15 Hz. (b,c) Absolute value of the low field (<0.2 MV/m) longitudinal electrostriction strain coefficient as a function of frequency for ceria ceramics with 10 mol % aliovalent dopants as calculated by linear regression. In (b), the fit curves for Debye-type relaxation as a function of frequency underlie the measured data points. In (a,b), only the upper error bar is shown to improve clarity. (d) Debye relaxation time τ (see Table 1) obtained by fitting data from (b) to eq 3.

All ceramic samples display electromechanical response at the second harmonic, a defining characteristic of electrostriction. Similar to previous reports on Sm- and Gd-doped ceria,^9,16,54^ the calculated electrostriction strain coefficients are considerably larger than those predicted by Newnham’s empirical scaling law^28,29^ for classical electrostrictors (≈10^–20^ m^2^/V^2^). As has been observed for other fluorite crystalline dielectrics, the ceria-based ceramic samples studied here contract in the direction parallel to the electric field (for convenience, the absolute values of strain and electrostriction strain coefficients are presented). Unlike the mechanical behavior described above, the frequency and electric field dependence of the electrostriction response differ substantially for ceramics with different dopants. There are striking differences in the electromechanical behavior between the smaller (Yb- and Lu-)doped ceramics and ceramics containing the larger dopants (Figure 4). Whereas the latter display strain saturation, the former do not (Figure 4a). (For details, see Section S2.)

For the electric field amplitude *E* ≤ 0.2 MV/m, *f* = 0.15 Hz, the electrostrictive strain is linearly proportional to *E*^2^ for all dopants
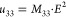
2where *M*_33_ is the longitudinal electrostriction strain coefficient. For all samples, *M*_33_ at low field exhibits varying amounts of decay with frequency between 0.15 and 150 Hz (Figure 4b,c). In the case of Nd-, Sm-, Gd-, and Er-doped ceria ceramics, the decay of *M*_33_ with frequency, *f*, can be reasonably fit to the non-ideal Debye relaxation equation with four parameters (Table 1)
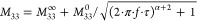
3where *M*_33_^∞^ and *M*_33_^0^ are parameters characterizing the electrostriction strain coefficient following and prior to relaxation, respectively, τ is the relaxation time (in sec), and α = 0 for the ideal case. Small values of α attest to a narrow distribution of relaxation times (cooperativity).^55^

**Table 1:** Fitting the Frequency Dependence of the Electrostriction Strain Coefficient of 10 mol % Aliovalent-Doped Ceria Ceramics to the Parameterized Non-ideal Debye Relaxation Equation  (Eq 2) Using the Levenberg–Marquardt Algorithm (Matlab)

dopant	τ, s	α	|*M*_33_^0^| × 10^–18^ m^2^/V^2^	|*M*_33_^∞^| × 10^–18^ m^2^/V^2^	*R*_adj_^2^
Er	1.1 ± 0.2	0^a^	54 ± 7	5.7 ± 0.3	0.9982
Gd	0.36 ± 0.03	0.11 ± 0.16	84 ± 3	1.0 ± 0.8	0.9990
Sm	0.24 ± 0.02	0.07 ± 0.07	88 ± 2	0.74 ± 0.71	0.9996
Nd	0.13 ± 0.01	1.0 ± 0.7	44 ± 2	0.5 ± 0.1	0.9938

a α was restricted to ≥0 during fitting (ideal = 0).

**Figure 5:**
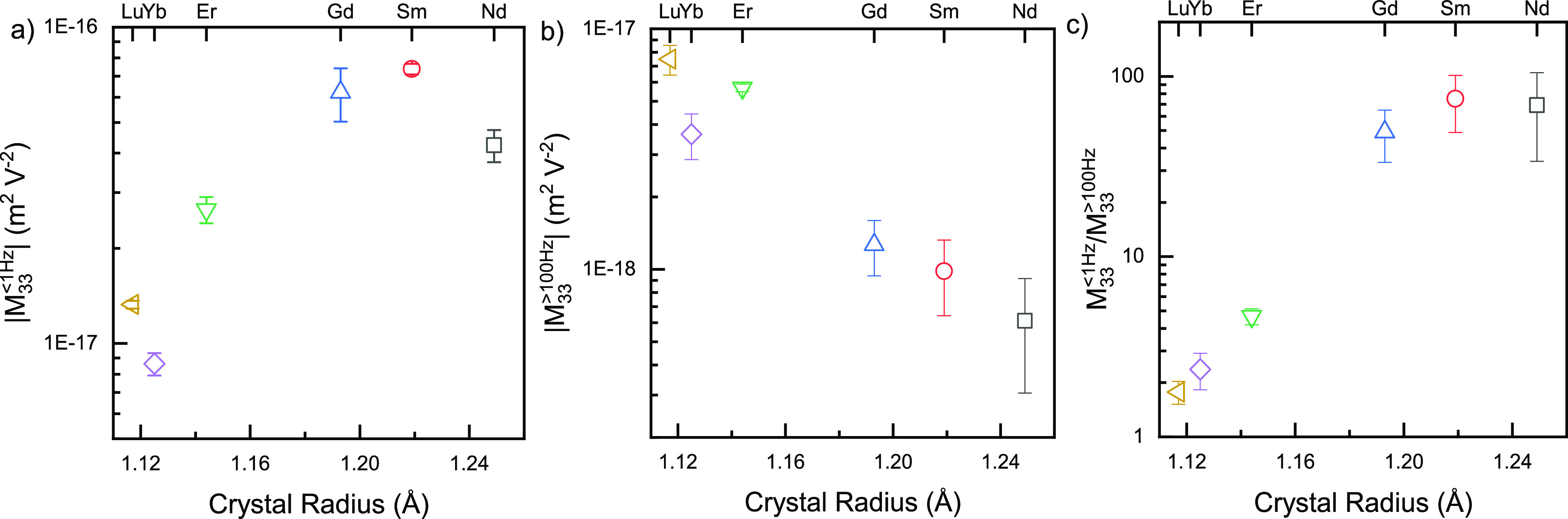
Semi-logarithmic graphs of the dependence of longitudinal electrostriction strain coefficients (|*M*_33_|) on dopant size for ceria ceramics with 10 mol % aliovalent dopants: (a) low-frequency (*f* < 1 Hz) average; (b) high-frequency (> 100 Hz) average; and (c) the ratio. In (a), error bars inside the symbols indicate that the uncertainty is smaller than the size of the symbol.

The relaxation time τ decays monotonically with the crystal radius of the dopant in the order Er, Gd, Sm, and Nd (Table 1), while in the case of Lu and Yb, *M*_33_(*f*) cannot be fit to eq 3. Therefore, to allow comparison of the extent of frequency relaxation for all samples, we used the average of the measured values *f* < 1 Hz, *M*_33_^<1Hz^, and *f* > 100 Hz, *M*_33_^>100Hz^, as an empirical measure (Figure 5a,b).

For Yb- and Lu-doped ceria, the decay with the frequency over the complete range investigated is relatively weak: the ratio *M*_33_^<1Hz^/*M*_33_^>100Hz^ < 3 (Figure 5c), whereas for Gd, Sm, and Nd, this decrease approaches 100-fold. The low frequency coefficient *M*_33_^<1Hz^ increases from Yb to Sm by almost an order of magnitude. By contrast, *M*_33_^>100Hz^ decreases with increasing crystal radius from Lu to Nd (Figure 5b). If we examine the ratio of the low- and high-frequency coefficients (Figure 5c), we find a marked difference between the three smaller dopants and the three larger dopants, similar to observations for IT ionic conductivity of ceria-based ceramics.^2,56^

The mechanical and electrostrictive properties of 10 mol % trivalent-doped dense ceria ceramics are summarized as follows:

Each of the 10 mol %-doped ceria ceramics shows room-temperature viscoelastic/anelastic behavior: Young’s moduli derived from slow (0.15 mN/s) indenter unloading are much larger than those determined with USTOF. However, while Gd-doped ceria and Sm-doped ceria display the largest values of the room-temperature primary creep constant, the creep constant for Nd-doped ceria cannot be distinguished from those determined for ceramics containing one of the three smaller cations.

Within the frequency range examined (0.150 mHz to 130 Hz), the longitudinal electrostriction strain coefficient for all ceramic samples (*M*_33_) is consistently ≥10^2^-fold larger than the value estimated on the basis of the classical scaling law, that is, ≈10^–20^ m^2^/V^2^.^9,20,28,30^ All samples exhibit some reduction of electrostrictive strain with frequency: ceramics containing Er, Gd, Sm, and Nd display Debye-type strain relaxation above ∼1 Hz, while Lu and Yb are considerably less sensitive to the increase in electric field frequency. In fact, the dependence of the averaged low-frequency electrostriction coefficients, *M*_33_^<1Hz^, on the dopant crystal radius is opposite to that of the averaged high frequency electrostriction coefficients, *M*_33_^>100Hz^. Such strikingly different behavior implies that lattice defects—oxygen vacancies and aliovalent dopants—may produce more than one type of polarizable elastic dipoles (see Supporting Information, Section S3) and that the crystal radius of the dopant may determine which dipole controls the ceramic response as a function of electric field frequency. The electrostriction relaxation time τ decreases monotonically from Er to Nd, revealing that the rate of reorientation of the elastic dipoles increases with the dopant crystal radius. The averaged low-frequency (*M*_33_^<1Hz^) electrostriction strain coefficients are found to follow the dopant–vacancy association energy,^1^ which increases as the dopant size decreases due to the increase in electrostatic attraction. This could explain the correlation with IT ionic conductivity (Supporting Information, Figure S5) and suggests that the type of elastic dipole defining electrostriction at low frequency may involve oxygen vacancies.

A model for the elastic dipole originating from the presence of oxygen vacancies in reduced ceria was proposed by Qi.^31^ DFT calculations showed that charge disproportionation among the four cerium atoms, tetrahedrally coordinated around an oxygen vacancy, produced asymmetric lattice distortion and the formation of an elastic dipole. This elastic dipole was found to be highly polarizable, which may explain its contribution to non-classical electrostriction. Reorientation of such an elastic dipole in an electric field would require simultaneous changes in oxygen vacancy–cerium distances by ≈0.014 nm, which may be the origin of the observed relaxation phenomenon.

Nevertheless, there is, as yet, no detailed model for the type of polarizable elastic dipole responsible for electrostrictive strain at high frequencies. This more rapid response appears not to depend on the extent of oxygen vacancy-induced elastic dipole orientational freedom and, by extension, also not on IT ionic conductivity; ceria ceramics containing the small dopants, Lu, Yb, and Er, are in fact poor ion conductors. Reverse Monte Carlo calculations^36^ did reveal that local lattice distortion in Y- and Sm-doped ceria can be explained by bimodal distributions of cation/anion bond lengths. Future models should certainly use this as a starting point. It is important to note that the technologically important frequency range for the application of electrostrictors is tens of Hz to tens of kHz. Since good IT ionic conductivity may produce non-homogeneous electric fields in the interior of a ceramic even at room temperature, the fact that the electrostrictive response of ceria solid solutions at high frequencies appears not to be related to ionic conductivity is promising for the development of practically useful electrostrictors. Whether further decrease of the dopant crystal radius will result in increased electrostrictive response at high frequencies requires further investigation.

## 4 Conclusions

The present study of the modulating effects of the aliovalent dopant size on the ceria-based ceramic electromechanical response is focused on the development of low dielectric constant, nontoxic, and environmentally friendly electrostrictors. We anticipate that continued theoretical (DFT)/modeling (reverse Monte Carlo) calculations, together with experimental data, will pave the way to simplifying the design of transducers for a broad range of devices, including actuators and sonars, but with the proviso that the amplitude of the high frequency strain response will be successfully increased. In view of our observation that the dopant crystal radius influences the ceramic electrostrictive response as a function of electric field frequency, we suggest that, in ceria solid solutions, the cubic lattice must be able to support at least two types of polarizable elastic dipoles, those undergoing strong strain relaxation above 1 Hz and those capable of responding to at least 100 Hz; the crystal radius of the dopant may determine which dipole controls the ceramic response as a function of electric field frequency.

## Supporting Information Available

Correction of elastic moduli for ceramic porosity; time decay of ultrasound pulse echo; uncorrected sound velocities; intrinsic and extrinsic anisotropic strain due to point defects in aliovalent-doped ceria: elastic dipoles; typical indenter displacement; saturation field and strain; and bulk ionic conductivity of ceria ceramic pellets doped with 10 mol % trivalent rare earth elements and measured at *T* = 673 K (PDF)
