# Supplementary material for: Trivalent Dopant Size Influences Electrostrictive Strain in Ceria Solid Solutions
Source: ACS Appl Mater Interfaces. 2021 Apr 22;13(17):20269–76. doi: 10.1021/acsami.0c20810 (PMC8288944; doi:10.1021/acsami.0c20810)
Supplement: Supplementary file 1 — am0c20810_si_001.pdf [file am0c20810_si_001.pdf]

Supporting Information

Trivalent dopant size influences electrostrictive strain in ceria solid solutions

Maxim Varenik[1], Juan Claudio Nino[2], Ellen Wachtel[1], Sangtae Kim[3], Sidney R. Cohen[4], and Igor Lubomirsky[1]

[1]Department of Molecular Chemistry and Materials Science, Weizmann Institute of Science, Rehovot, 761001, Israel

[2]Department of Materials Science and Engineering, University of Florida, Gainesville, Florida, 32611, United States

[3]Department of Materials Science and Engineering, University of California, Davis, Davis, California, 95616, United States

[4]Dept. Chemical Research Support, Weizmann Institute of Science, Rehovot, 761001, Israel

Corresponding Author
Igor Lubomirsky. Department of Molecular Chemistry and Materials Science
Weizmann Institute of Science, Rehovot 761001, Israel;
orcid.org/0000-0002-2359-2059;
Email: igor.lubomirsky@weizmann.ac.il

Supporting Information

Section 1. Correction of elastic moduli for porosity [1-2]

Shear (G_0 or C_{44}) and longitudinal (C_{11}) moduli were calculated from the corresponding sound velocities:

(Eqs. S1) $$C_{11} = \rho_m \cdot V_L^2 \; and \; G_0 = \rho_m \cdot V_S^2$$

where ρ_m is the measured pellet density. The measured sound velocities are reported in Figure S2. These moduli were used to calculate the Young's (E_0) and bulk (B_0) moduli, and Poisson's ratio (υ_0):

(Eqs. S2) $$E_0 = G_0 \frac{3V_L^2 - 4V_S^2}{V_L^2 - V_S^2}, \upsilon_0 = \frac{E_0}{2G_0} - 1, B_0 = \frac{E_0 \cdot G_0}{3(3G_0 - E_0)}.$$

The dynamic model (Ledbetter *et al.* [1-2]) was used to correct for porosity (p) :

(Eqs. S3) $$G_D = \frac{-F + \sqrt{F^2 - 4AC}}{2A} \quad \text{and} \quad B_D = B_0 \frac{4G_D}{4(1-p) \cdot G_D - 3p \cdot B_0}$$

where $A = 8\frac{1-p}{3}$; $C = -3G_0 \cdot B_0(1+p)$; $F = (3-2p) \cdot B_0 - (8/3 + 4p) \cdot G_0$ .The subscript "0" denotes the values of the elastic moduli before correction for porosity as calculated using Eqs. S1, while the subscript "D" denotes the values corrected for porosity according to the dynamic model. The approximate porosity limit for reliable correction is 6 vol% of the ceramic. As far as the influence of porosity on electromechanical response is concerned, no effect of porosity < 10 vol% is anticipated, as only above this concentration might pores produce sufficient grain boundary space charge that would affect electrical or electromechanical properties. [3-4]

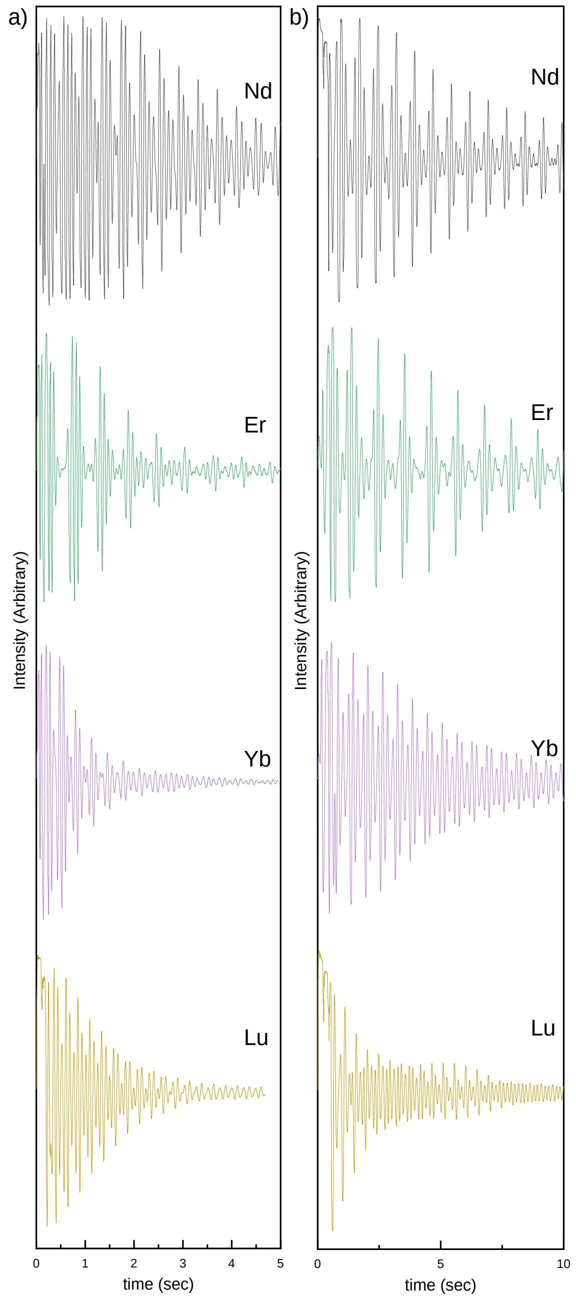

Figure S1. Time decay of ultra-sound pulse echo: (a) longitudinal; (b) shear. From these graphs, the possibility of microcracks in the ceramics is eliminated, which would not have

been possible using other techniques. Analogous data for Gd and Sm data have already been published.[5-6]

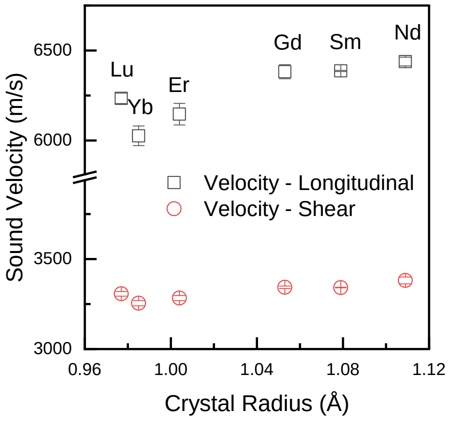

Figure S2. Longitudinal and shear sound velocities, uncorrected for pellet porosity, as obtained with ultra-sound pulse echo measurements.

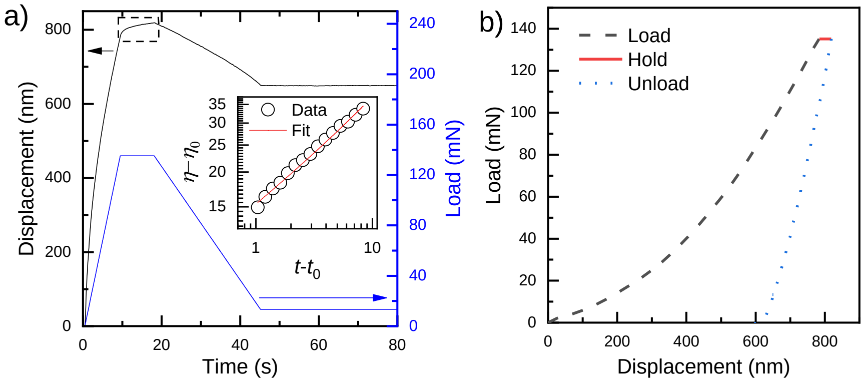

Figure S3. Indenter displacement (left, black) and load on sample (right, blue) during a fast nanoindentation measurement on a 10mol% Nd doped ceria pellet at room temperature; primary creep is observed in the maximum load-hold phase (dashed rectangle in (a), and the graph inset of the matching power law fit of 1/3 (red line). Creep is also observed in load-displacement curves (b) as an increase in displacement at maximum load.

Section 2. Field and strain saturation for Er, Gd, Sm and Nd doped ceria ceramics

At $f \leq 1$ Hz and $E > 0.2$ MV/m, the dependence of longitudinal strain, u_{33}, on the applied field (Figure 4a) can be approximated as:

(Eq. S1) $$u_{33} = u_{sat}\left(1 - e^{-E^2/E_{sat}^2}\right)$$

where E_{sat} is the electric field at which strain saturates and u_{33} is the saturation strain. Sm doped ceria presents the smallest values of E_{sat} and u_{sat} within the frequency range studied while Er presents the largest values of E_{sat} and Er and Gd, the largest values of u_{sat}. (Figure S4). Technical limitations prevent saturation strain from being measured at frequencies above 1 Hz. To date, no unambiguous explanation of the observed saturation strain has been found. However, the polarizable elastic dipole model is consistent with this observation. We may speculate that reorientation of elastic dipoles under electric field will saturate when all dipoles are aligned parallel to the field , *i.e.*, in analogy to saturation magnetization observed for paramagnetic materials.

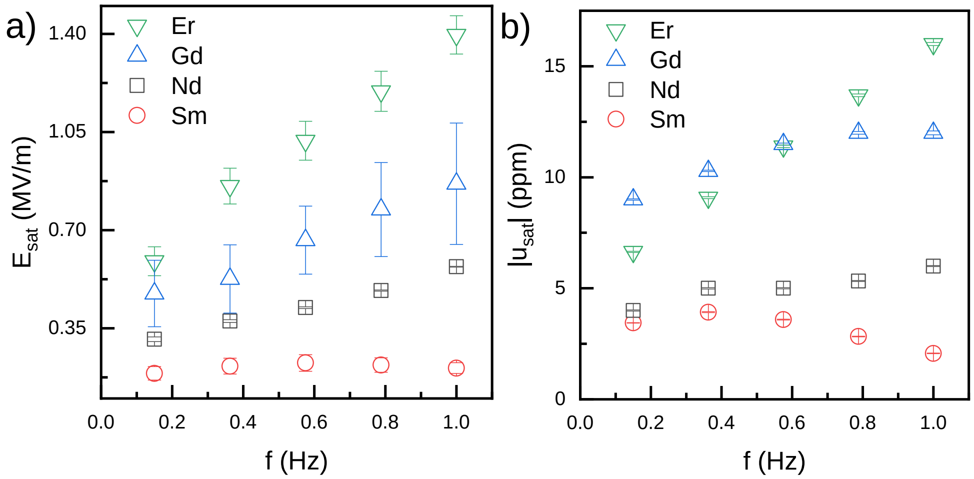

Figure S2. (a) Saturation field (E_{sat}) and (b) longitudinal saturation strain (u_{33}), calculated according to Eq. S4, for $RE_{0.1}Ce_{0.9}O_{1.95}$ (RE = Er, Gd, Sm, and Nd) ceramics as a function of frequency for 0.15 Hz ≤ f ≤ 1 Hz. Yb and Lu ceramics do not display saturation strain. Interior error bars indicate measurement uncertainty smaller than the size of the symbol.

Section 3. Intrinsic and extrinsic anisotropic strain in reduced/aliovant doped ceria: elastic dipoles

To date, single crystals of reduced or aliovalent doped ceria (CeO_2), of sufficient size to permit measurement of the directional dependence of elastic moduli, are not available. However, it is well known that other inorganic, crystalline dielectrics with fluorite *(Fm-3m)* symmetry (*e.g.,* CaF_2) display anisotropic mechanical properties in spite of the fact that the dielectric properties are not anisotropic. The absolute value of the elastic compliance tensor (**S**) for CaF_2 is maximum in the <111> direction and minimum parallel to the axes (*e.g.,* <100>) of the cubic unit cell [7]. From these data we learn that even defect-free cubic crystalline dielectrics may display anisotropic mechanical properties, and, consequently, anisotropic electrostrictive strain as well.

For doped or reduced ceria, it is necessary to consider the effect of the introduction of point defects into a fluorite crystal lattice which is (already likely to be) mechanically anisotropic. Two types of point defects can be identified in reduced or aliovalent-doped ceria: (1) vacant oxygen sites and (2) aliovalent dopant cations (III) with ionic radius either larger or smaller than that of the cerium (IV) host. Two dopant cations are required to provide charge-compensation for each oxygen vacancy. A point defect in an elastic medium may be modelled as an elastic dipole.[8-9]

In elasticity theory, an elastic dipole (P_{jk}) is defined as the first moment (N.B., an elastic monopole does not exist) of the distribution of point forces:

(Eq. S5) $P_{jk}=\Sigma F_j^q \, a_k^q$,

where *q* indexes the number of forces; F is a force and a is a displacement . P_{jk} is a second rank tensor. Viewing a point defect as an elastic dipole allows us to characterize its long

range elastic field as well as its interaction with other sources of stress. The local elastic displacements, caused by the point defect, decay as $1/r^2$ and the intrinsic stress, as $1/r$, [8-9] where r=0 locates the defect itself. The energy of interaction (**E_{int}**) of the elastic dipole with externally applied sources of stress takes the form:

(Eq. S6) $E_{int} = -P_{ij}\, u_{ij}^{ext}(0)$,

where $u_{ij}^{ext}(0)$ is the resultant strain at the site of the point defect. To add additional complexity, if the point defect can adopt different configurations, then the occupancy of these configurations will be modified under anisotropically applied stress or strain. The slow redistribution of the configurations of the point defect gives rise to anelasticity, *i.e.* time dependent mechanical response.[10] In order for the elastic dipole to interact with an externally applied electric field, changes in bond lengths (*e.g.* the Ce-O bonds) must be electrically polarizable.

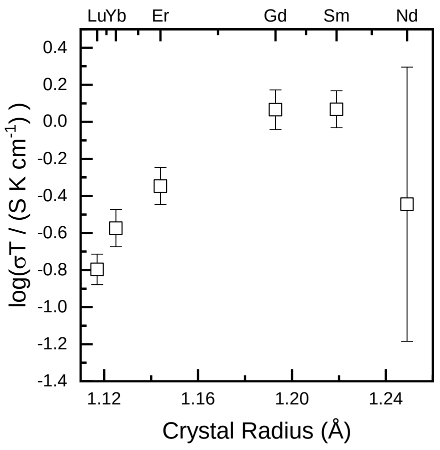

Figure S5. Bulk ionic conductivity of ceria ceramic pellets doped with 10mol% trivalent rare earth elements and measured at T=673K.[11]

REFERENCES

(1) Ledbetter, H. M.; Austin, M. W.; Kim, S. A.; Lei, M. Elastic Constants and Debye Temperature of Polycrystalline $Y_1Ba_2Cu_3O_{7-x}$. J. Mater. Res. 2011, 2 (6), 786-789, DOI: 10.1557/JMR.1987.0786.

(2) Ledbetter, H. M.; Datta, S. K. Effective Wave Speeds in an Sic-Particle-Reinforced Al Composite. J. Acoust. Soc. Am. 1986, 79 (2), 239-248, DOI: 10.1121/1.393565.

(3) Pérez-Coll, D.; Sánchez-López, E.; Mather, G. C. Influence of Porosity on the Bulk and Grain-Boundary Electrical Properties of Gd-Doped Ceria. Solid State Ionics 2010, 181 (21-22), 1033-1042.

(4) Jo, S. H.; Muralidharan, P.; Kim, D. K. Electrical Characterization of Dense and Porous Nanocrystalline Gd-Doped Ceria Electrolytes. Solid State Ionics 2008, 178 (39-40), 1990-1997.

(5) Yavo, N.; Noiman, D.; Wachtel, E.; Kim, S.; Feldman, Y.; Lubomirsky, I.; Yeheskel, O. Elastic Moduli of Pure and Gadolinium Doped Ceria Revisited: Sound Velocity Measurements. Scr. Mater. 2016, 123, 86-89, DOI: 10.1016/j.scriptamat.2016.05.029.

(6) Varenik, M.; Cohen, S.; Wachtel, E.; Frenkel, A. I.; Nino, J. C.; Lubomirsky, I. Oxygen Vacancy Ordering and Viscoelastic Mechanical Properties of Doped Ceria Ceramics. Scr. Mater. 2019, 163, 19-23, DOI: 10.1016/j.scriptamat.2018.12.024.

(7) Newnham, R. E.; Sundar, V.; Yimnirun, R.; Su, J.; Zhang, Q. M. Electrostriction: Nonlinear Electromechanical Coupling in Solid Dielectrics. J. Phys. Chem. B 1997, 101 (48), 10141-10150, DOI: 10.1021/Jp971522c.

(8) Clouet, E.; Varvenne, C.; Jourdan, T. Elastic Modeling of Point-Defects and Their Interaction. Comp. Mater. Sci. 2018, 147, 49-63.

(9) Varvenne, C.; Clouet, E. Elastic Dipoles of Point Defects from Atomistic Simulations. Phys. Rev. B 2017, 96 (22), 224103.

(10) Nowick, A. S.; Berry, B. S. Anelastic Relaxation in Crystalline Solids, Academic Press: New York, 1972.

(11) Koettgen, J.; Grieshammer, S.; Hein, P.; Grope, B. O. H.; Nakayama, M.; Martin, M. Understanding the Ionic Conductivity Maximum in Doped Ceria: Trapping and Blocking. Phys. Chem. Chem. Phys. 2018, 20 (21), 14291-14321, DOI: 10.1039/c7cp08535d.
